# Effects of Individual and Pair Housing of Calves on Short-Term Health and Behaviour on a UK Commercial Dairy Farm

**DOI:** 10.3390/ani13132140

**Published:** 2023-06-28

**Authors:** Sophie A. Mahendran, D. Claire Wathes, Richard E. Booth, Neil Baker, Nicola Blackie

**Affiliations:** 1 Royal Veterinary College, Pathobiology and Population Sciences, Hawkshead Lane, Hatfield AL9 7TA, UK; dcwathes@rvc.ac.uk (D.C.W.); rbooth@rvc.ac.uk (R.E.B.); nblackie@rvc.ac.uk (N.B.); 2 Leaze Farm, Haselbury Plucknett, Crewkerne TA18 7RJ, UK

**Keywords:** calf housing, pair, individual, health, behaviour, non-nutritive abnormal oral behaviour

## Abstract

**Simple Summary:**

The impact of pair compared to individual housing of pre-weaning calves has previously demonstrated benefits in terms of socialization and calf development. This study aimed to investigate the impacts of individual and pair housing on a UK commercial dairy farm and establish further behavioural impacts that housing groups can have on calves. Overall, we found an increase in the activity of pair-housed calves, with individually housed calves spending more time with their head out of the front of the pen, as well as spending longer engaged in self-grooming. There was no impact of housing type on average daily liveweight gain, but there was an increase in disease prevalence in individually housed calves, possibly due to the impact of stress induced by social isolation. Overall, pair housing had a positive impact on the health and behaviour of calves on a commercial UK dairy farm.

**Abstract:**

Social pair housing of calves has previously demonstrated positive impacts for calves, so this study aimed to compare the health and behaviour of calves kept in individual compared to pair housing on a single commercial UK dairy farm. A total of 457 Holstein and Jersey heifer calves were recruited and systematically allocated to individual and pair housing. Weekly visits were conducted up to 8 weeks of age, with weight and presence of clinical disease measured using both a standardized scoring system and thoracic ultrasonography. A subset of calves (*n* = 90) had accelerometers attached to monitor activity, with CCTV placed above a further 16 pens to allow behavioural assessments to be made via continuous focal sampling at 1 and 5 weeks of age. During the study, there was a mortality rate of 2.8%, and an average daily liveweight gain (ADLG) of 0.72 kg/day, with no significant effect of housing group (*p* = 0.76). However, individually housed calves had increased odds of developing disease (OR = 1.88, *p* = 0.014). Accelerometer data showed that housing group had no effect on lying times, with a mean of 18 h 11 min per day (SD 39 min) spent lying down. The motion index was significantly higher in pair-housed calves (F_1,83_ = 440.3, *p* < 0.01), potentially due to more social play behaviour. The total time engaged in non-nutritive oral behaviours (NNOBs) was not impacted by housing group (*p* = 0.72). Pair-housed calves split their time conducting NNOBs equally between inanimate objects and on their pen mates’ body. Individually housed calves spent significantly more time with their head out of the front of the pen (*p* = 0.006), and also engaged in more self-grooming than pair-housed calves (*p* = 0.017), possibly due to a lack of socialization. The overall findings of this study indicate that within a UK commercial dairy management system, pair-housed calves were healthier and more active than individually housed calves, while housing group did not influence ADLG or the occurrence of NNOBs.

## 1. Introduction

The housing management of pre-weaning calves strongly impacts their health and behavioural development. Traditional farming techniques utilized individual housing with the aim of bio-containing neonatal pathogens linked to bovine respiratory disease (BRD) and diarrhoea [1,2]. This individual housing choice is still prevalent across North America [3] and Europe [4,5] due to stock people’s concerns over disease transmission [6], potential cross sucking and health monitoring [7]. However, the importance of socialization for calves is now more thoroughly understood, with social facilitation and learning [8] playing a key role in reducing neophobia [9] and increasing solid feed intakes [10,11,12,13,14]. Socialization can be provided by pair or group housing, with pair housing having the benefit of still limiting the overall amount of calf–calf contact for reduced transmission of disease [15] and easy disease monitoring [16]. Indeed, disease levels in pair housing systems have been shown to remain similar to those of individually housed calves [17,18,19,20,21,22].

Social isolation can result in the development of abnormal behaviours such as excessive licking of a calf’s own body or pen fixtures [23]. These abnormal oral behaviours (OBs) are commonly recorded in other species such as equines [24], and occur as a result of domestication, with modern management practices limiting the ability of animals to fulfil their natural behaviour patterns. Abnormal OBs have been recognized in veal calves for many decades [25,26], with the low level of environmental stimulation in individual pens resulting in calves developing their own self-directed activities. The licking of pen fixtures may be redirected social grooming [27], although licking frequency is also affected by environmental factors, with calves housed indoors as opposed to outside showing more licking behaviours [28]. Pen licking can also be influenced by nutrition levels, with more pen licking observed in the period prior to weaning when feed intake is insufficient to stimulate significant amounts of rumination [29].

Excessive self-grooming by calves can also be an abnormal OB, with lower levels recorded in pair compared to individually housed calves [17]. Self-grooming enables hygienic maintenance of a calf’s body, which would normally be carried out by the dam [30], but it can also be influenced by the bedding substrate, with sand-bedded calves spending more time grooming than those on straw [31]. Levels of self-grooming are reduced in diseased calves [32], which suggests a baseline level is normal and potentially a good indicator of calf welfare. However, excessive levels of self-grooming turn the behaviour into an abnormal oral behaviour (AOB), and again represent redirected social grooming.

In addition to licking and grooming, abnormal sucking behaviours are commonly reported in social housing situations, representing a non-nutritive oral behaviour (NNOB) [33]. NNOBs are often termed cross sucking or navel sucking when they are performed by one calf on another, with the majority of sucking directed at the ventral region (especially the udder or scrotum) of other calves [29]. NNOBs are thought to occur due to an accumulation of internal motivation to perform specific motor patterns [34], in this case sucking to consume milk. This is why restrictive milk feeding regimes or restricted time being allowed to suck on a teat (particularly by bucket feeding milk) are associated with NNOBs, but they are rarely observed in dam-reared calves. NNOBs have their highest frequency of occurrence 1 min after the end of milk-feeding, declining to negligible levels by 13 min post-milk feeding [29]. NNOBs can become problematic if they lead to hair loss and inflammation of the sucked area [35], but can be decreased when calves are allowed to redirect their sucking motivation to a dry teat on the wall [36].

Although there is a wide range of evidence favouring pair housing systems, many studies have been conducted on research facilities outside of the UK, predominantly in Canada and the USA, which generally experience much colder winters and higher temperature humidity indexes (THI) during the summer periods compared to the UK. Given that environmental conditions can impact both health and behaviour of calves, establishing the impact of individual and pair housing under UK weather conditions is important. Much of the published literature also originates from research units rather than commercial dairy farms, so the applicability of findings is questionable due to differences in management and financial motivations for daily calf care. Given these differences, this study aimed to assess the impact of individual and pair housing of calves on a commercial UK farming system to establish the short-term impacts on calf health and behaviour.

## 2. Materials and Methods

### 2.1. Animals and Housing

This study was approved by the Clinical Research Ethics Committee of the Royal Veterinary College (protocol code URN SR2019-0369, 27/03/20). It was conducted on a single commercial dairy farm in the south-west of England from March to December 2020. The farm milked 1800 Holstein and Jersey dairy cows in an all-year-round calving pattern. Calves were born in a loose-housed straw yard and were provided with two 3 L colostrum feeds from their own dam within 12 h of birth via an oesophageal feeding tube. Calves were transported two miles to a rearing site, where they were housed in specially built calf sheds, with pens formed from pre-fabricated plastic dividers (Calf-Tel Pen system, Hampel Corp, Germantown, WI, USA), with internal dimensions 122 cm × 213 cm for an individual pen, and twice this for a pair pen. Pens were arranged side by side, with tactile contact possible over the top of pen dividers, and front openings to allow the head out of the pen. Calves were systematically allocated at birth into housing groups of either individuals or pairs.

### 2.2. Calf Nutrition

Each calf was fed a 24% whey protein and 20% fat calf milk replacer (Maximise, Nukamel, SE Weert, The Netherlands) mixed at 15% concentration, given at 3 L twice daily through a teat feeder. The calves were weaned from 7 weeks of age by reducing milk volume daily until they came off milk at 8 weeks of age. Each pen had ad libitum water from a bucket. Calves were provided with ad libitum pelleted concentrate, with 18% crude protein, 4% fats and 12% crude fibre (Super Rearer 18 Nuts, ForFarmers, Bury St Edmonds, UK). From four weeks of age, calves were provided with an ad libitum total mixed ration with grass silage, chopped wheat straw, caustic wheat, mineral mix and rapeseed meal, with an overall content of 22% crude protein and 20% starch. 

### 2.3. Performance and Health

Eight visits over consecutive weeks were conducted for each calf by the researcher (SAM). At the first visit between 1 and 8 days of age, a jugular blood sample was collected in a plain vacutainer as part of the farm’s standard management protocol. The samples were left to stand for 24 h, before a sample of serum was placed onto a refractometer to assess serum total protein (TP).

At each weekly visit, the calf weight was estimated using a weigh band (AHDB, Stoneleigh Park, Warwickshire, UK) placed around the heart girth circumference to allow growth rate calculations. The average growth rate over the entire pre-weaning period was calculated, as well as over three time periods: 1 to 3 weeks, 4 to 6 weeks and 7 to 8 weeks. This allowed for compatible comparisons between all calves, regardless of the exact age at each measurement [20].

At each weekly visit, the calves underwent a clinical health assessment following the Wisconsin calf health scoring system [37] to assess demeanour, nasal and ocular discharge, cough, faecal consistency, rectal temperature and navel and joint health. A diagnosis of bovine respiratory disease (BRD) was given when a calf displayed at least one upper respiratory sign (nasal/ocular discharge or cough at score 2) along with pyrexia (≥39.5 °C). A diagnosis of diarrhoea was made when a faecal score of 3 was given (watery, sifts through bedding), with a diagnosis of navel ill and joint ill made when a score of 2 was given (swelling with pain or heat). There was no additional data collection conducted between visits due to reduced reliability of farm records compared to researcher-collected data. Calves identified as ill were treated according to standard veterinary practices by farm staff. When the variable of ‘disease’ was analysed, it was used as a binary variable, combining all causes of disease together.

Thoracic ultrasonography of calves was carried out at seven weeks of age. After application of 70% isopropyl alcohol to each thoracic area, a 7.5 MHz linear transducer was used to assess both sides of the thoracic cavity for pathology [38]. A categorical scoring system was used to record lesions, where Score 0 indicated normal aerated lungs with none to a few comet tail (B-line) artefacts, Score 1 indicated diffuse comet tails but without consolidation and Score 2 indicated lobular or patchy pneumonia with consolidation [39].

### 2.4. Novel Object Approach

An open umbrella was used as a novel object, which was placed into each calf pen during the sixth visit. Prior to placement, it was ensured that the calves were standing up within the pen. The time was measured from the placement of the umbrella until it was touched by the nose of a calf. In pair pens, the time was stopped when just one of the calves made contact with the umbrella. The calves were observed for a maximum time of 10 min, and if no contact was made, it was recorded as a non-approach [20].

### 2.5. Calf Activity

Automatic data logger tri-axial accelerometers (IceQube IceRobotics Ltd., South Queensferry, UK) were fitted to a hind leg of a subset of calves (*n* = 90) within one week of age. These data loggers measured the lying and activity times of calves (via calculation of motion index, which is a measure of the intensity of movement), and have been validated for use in calves [40]. These accelerometers were removed following the weaning of the calf.

### 2.6. Calf Behaviour

Video cameras (Hik-Vision, Hangzhou, China) were placed above pens on one side of a calf barn, allowing a subset of 16 pens of calves (8 individually housed and 8 pair-housed) to be recorded during the pre-weaning period. Continuous focal sampling from the video footage was conducted using Behavioral Observation Research Interactive Software (BORIS, Leeds, UK, version 7.9.6, [41]), with calves observed for 48 h at both one week and five weeks of age. The video footage was assigned random numbers using a research randomiser to enable observations to be carried out in a random number order. Once the data were captured, the identifying name for each video was entered. During the video observations, the behaviour of the calves was recorded according to Table 1. It should be noted that it was not possible to blind researchers to the housing group whilst video observations were conducted, meaning bias could not be eliminated. 

Calves were disbudded between 4 and 5 weeks of age by farm staff using local anaesthetic and non-steroidal anti-inflammatory drugs.

### 2.7. Statistical Analysis

This study formed part of a larger project assessing the long-term impact of calf housing up until the end of first lactation, with a sample size calculation based on identifying a 500 L milk yield difference in the 305-day milk yield of heifers, taking into account that approximately 30% of heifers do not reach the end of their first lactation. Using a 2-tailed test, a variance of 0.10, a confidence level of 0.95 and a power of 0.8, the sample size for detecting a significant difference was 150 individually housed and 300 pair-housed calves (150 pairs of calves).

Linear mixed effects models were used to assess the outcomes of average daily liveweight gain (ADLG) over the three time periods, the mean lying times and motion index of calves, and the differences in time budget of behavioural variables (lying position, NNOBs, self-grooming). The fixed effects included were month of enrolment, housing group (individual or paired), birthweight, breed, total protein level, disease presence, age at weaning and ultrasound score. The pen and calf identification number were included as random effects. The results are reported as F-values in the format F_(numerator df, denominator df)_. For all analyses, the assumption of normality was assessed through visual inspection of residual plots.

The outcome of disease occurrence was analysed using binary logistic generalised estimating equations, with the variable pen used to account for repeated measures within a pair of calves. The dependent variables were month of enrolment, housing group (individual or pair), birthweight and total protein level. The outcome of ultrasound score was analysed using ordinal logistic regression, with the variables of month of enrolment, housing group, breed, disease occurrence, total protein and ADLG. The outcome of novel object approach time was analysed using a generalised linear model, with the variables of month of enrolment, housing group and breed.

## 3. Results

### 3.1. Mortality

A total of 457 calves were recruited into the study over a nine-month period, with 26 Jersey and 421 Holstein heifers. During the study, 13 calves died or were euthanized, giving an overall 2.8% mortality rate (Table 2). This left 164 individually housed calves and 280 pair-housed calves (140 pairs).

### 3.2. Birthweight

The average birthweight of the calves was 37.7 kg (range 20.8–56.4 kg), and this had a significant effect on the ADLG (F_1,1114_ = 5.40; *p* = 0.02), with a 1 kg increase in birthweight resulting in a 9.0 g increase in ADLG. However, the breed was not found to be significantly associated with the ADLG (F_1,1114_= 1.268; *p* = 0.26), even though the mean birthweight of Holstein calves was 38.3 ± 0.22 kg and Jersey calves was 27.1 ± 0.60 kg. There was no significant effect on the ADLG of the month of enrolment (F_7,1114_ = 0.91; *p* = 0.50)*,* passive transfer (measured as serum TP) (F_1,1114_ = 1.93; *p* = 0.66), the occurrence of disease (F_1,248_ = 0.49; *p* = 0.48), the thoracic ultrasound score for the calf (F_2,1114_ = 0.067; *p* = 0.94) or the age at weaning (F_1,1114_ = 0.35; *p* = 0.55).

### 3.3. Weight Gain

**Figure 1:**
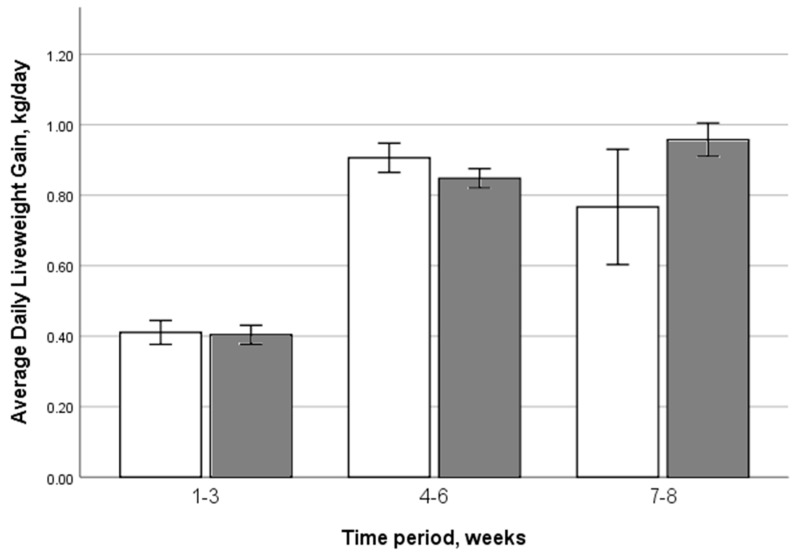
Assessment of the effect of pre-weaning housing group on the ADLG of the calves within three separate time periods prior to weaning. The mean ADLG in weeks 1–3 was 0.41 ± 0.1 kg/day (SEM), in weeks 4–6 was 0.87 ± 0.01 kg/day and in weeks 7–8 was 0.89 ± 0.03 kg/day. White columns indicate individually housed calves and grey columns indicate pair-housed calves.

The overall pre-weaning ADLG was 0.72 kg/day (range 0.29–1.09 kg/day). There was no significant association between housing group and the ADLG of the calves within the three separate time periods prior to weaning (F_1,1114_ = 0.091, *p* = 0.76; Figure 1). There was a non-significant numerical difference over the later pre-weaning period (weeks 7 to 8), with individually housed calves achieving 0.77 ± 0.08 kg/day (SEM) and pair-housed calves achieving 0.96 ± 0.02 kg/day. This suggested a tendency for pair-housed calves to have a greater average increase in weight gain as they got older compared to individually housed calves.

### 3.4. Passive Transfer and Disease Occurrence

The total protein levels taken in the first week of life ranged from 4.3 to 8.6 g/dL, with 95% of calves classed as having good passive transfer as indicated by a level of ≥5.2 g/dL [34], and no difference between the two housing groups. 

A total of 154/444 calves (34.7%) experienced disease during the pre-weaning period, with BRD being the most prevalent disease overall (Table 3). There was a significant effect of housing group (*p* = 0.014), with 41.5% of individually housed and 30.7% of pair-housed calves experiencing disease. Individually housed calves had an increased odds of developing disease (OR = 1.88 (1.14–3.12)).

The month of enrolment also had a significant effect on the occurrence of disease (*p* = 0.03), with calves born in the spring or summer less likely to suffer from disease compared to those born in the winter. There was no association between the total protein level (odds ratio (OR) = 0.99 (0.96–1.02); *p* = 0.52) or ADLG (OR = 1.14 (0.70–1.87); *p* = 0.59) and the occurrence of disease. Birthweight demonstrated a tendency for an association with the occurrence of disease (OR = 0.96 (0.92–1.01); *p* = 0.085), with calves that became ill having a lower median birthweight compared to their healthy counterparts (37.5 kg vs. 38.4 kg).

The thoracic ultrasound score taken during the seventh week of life demonstrated that 160/444 (36.0%) calves received a score of 1 (diffuse comet tails but without consolidation) and 27/444 (6.1%) received a score of 2 (lobular or patchy pneumonia with consolidation). There was no association between thoracic ultrasound scores and housing group (*p* = 0.44), breed (*p* = 0.65), ADLG (*p* = 0.18), occurrence of disease (*p* = 0.23), month of enrolment (*p* = 0.071) or total protein level (*p* = 0.82). 

### 3.5. Activity

**Figure 2:**
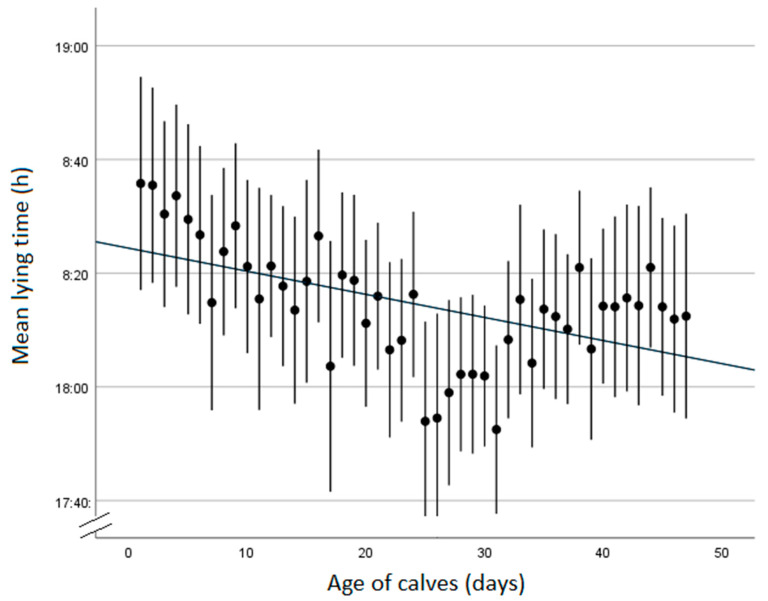
Mean lying times of calves over time, showing a reducing trend. Error lines are two standard errors, with a line of best fit. Calves were disbudded around 28–35 days of age, which may account for the reduced mean lying times seen at this time.

A total of 90 calves had accelerometers fitted, with 23 on individually housed calves and 67 on pair-housed calves. The mean daily lying time was 18 h 11 min per day (SD 39 min), with no association of the housing group (F_1,3_ = 0.19, *p* = 0.69), and an overall trend in reducing lying times as the calves got older (Figure 2). The birthweight of the calves was significantly associated with the mean daily lying time (F_3,79_ = 7.8, *p* = 0.007), with a 1 kg increase in birthweight corresponding to a reduction of 121 s in mean daily lying time. The month of birth was significantly associated (F_3,24_ = 3.1, *p* = 0.044), with calves born in July and August having longer lying times than those born in September and October (18 h 19 min vs. 17 h 47 min). The occurrence of disease was also significantly associated with the mean daily lying time (F_1,79_ = 6.3, *p* = 0.014), with calves suffering from illness having a longer lying time than their healthy counterparts (18 h 19 min vs. 18 h 6 min).

**Figure 3:**
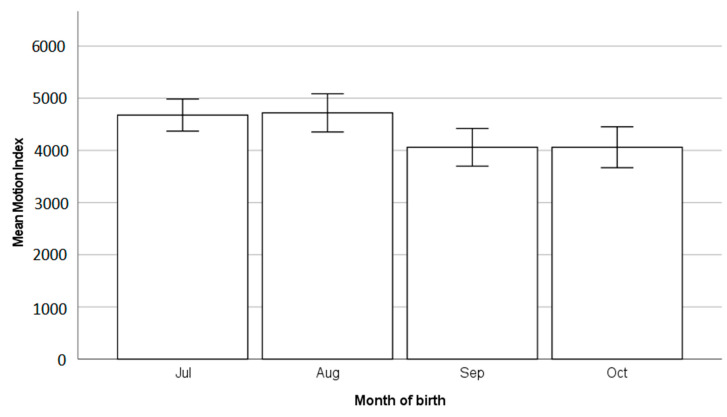
Mean motion index (MI) of calves over time, showing a reduction in MI later in the year. Error lines are two standard errors.

The motion index (MI, measure of intensity of activity) was significantly associated with the housing group (F_1,83_ = 440.3, *p* < 0.01), with pair-housed calves having a higher mean value of 4503.6 ± 117.5 compared to 4388.0 ± 179.2 in individually housed calves. The MI was significantly associated with the month of enrolment (F_1,83_ = 3.5, *p* = 0.019, Figure 3), and demonstrated a trend with the occurrence of any disease (F_1,83_ = 3.0, *p* = 0.088), with diseased calves having a lower motion index of 4137.2 ± 153.5 compared to non-diseased calves 4678.5 ± 120.7. The MI was not associated with the breed of the calf (F_1,83_ = 2.4, *p* = 0.13).

### 3.6. Behaviour

**Figure 4:**
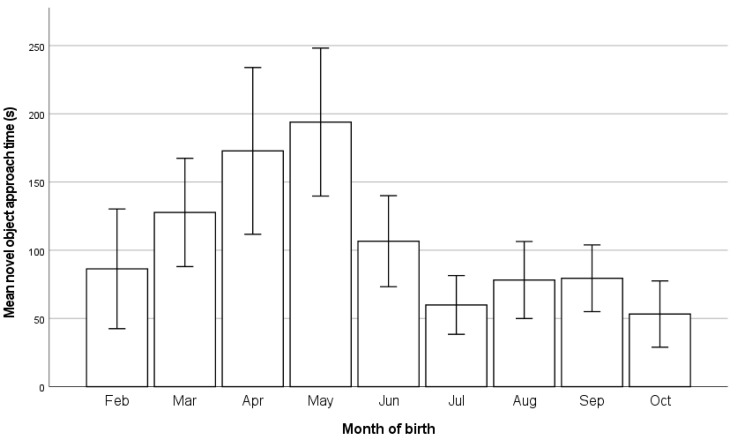
Mean novel object approach time of calves by month of birth. Error lines are two standard errors.

The novel object approach test was performed during the visit in week six. Of all the calves observed, 8/444 (1.8%) individually housed and 15/444 (3.4%) pair-housed calves did not approach the novel object within the observation time limit of 10 min. Of the calves that did approach, there was a significant effect of housing group (*p* < 0.01), with the mean time to approach being 84.0 ± 9.4 s (SEM) for individually housed calves and 121.2 ± 9.2 s (SEM) for pair-housed calves. The month of enrolment was significantly associated with the time to approach the novel object (*p* < 0.01), Figure 4. The breed was significantly associated with the time to approach (*p* < 0.01), with the Holsteins approaching in a mean 113.3 ± 7.1 s, and Jerseys in a mean 11.8 ± 2.4 s.

Behavioural assessments were conducted on video footage from eight individually housed calves and eight pairs of calves. The total time calves spent performing NNOBs was not associated with the type of housing (F_1,15_ = 0.13, *p* = 0.72), but was significantly associated with the age of the calves (F_1,32_ = 27.1, *p* < 0.01), with one-week-old calves spending a mean of 33 min and five-week-old calves a mean of 50 min per day performing NNOBs. Although there was no difference between the total time spent per day engaged in NNOBs, individually housed calves could only suck, lick or chew on inanimate objects in the pen, whereas the pair-housed calves split the same time between inanimate objects, the ventral area of their pen mate and other parts of their pen mate (Table 4).

The amount of time the calves spent with their head out of the front of the pen was significantly associated with the housing group (F_1,14_ = 10.6, *p* = 0.006), with individually housed calves spending a mean of 53 min/d and pair-housed calves spending a mean of 32 min/d with their heads out of the front of the pen. It was also significantly associated with the age of the calves (F_1,30_ = 22.0, *p* < 0.01), with one-week-old calves spending a mean of 34 min/d and five-week old calves a mean of 52 min/d with their head out of the front of the pen. 

The amount of time calves spent self-grooming was significantly associated with the housing group (F_1,10_ =8.2, *p* = 0.017), with individually housed calves spending a mean of 33 min/d and pair-housed calves spending a mean of 19 min/d self-grooming. Self-grooming was significantly associated with the age of the calves (F_1,29_ = 16.6, *p* < 0.001), with one-week-old calves spending a mean of 19 min/d and five-week-old calves a mean of 33 min/d self-grooming.

The assessment of the lying position of the calves within a pair demonstrated that calves spent most of their joint lying time within one calf length of each other (Table 5), with a significant increase in the amount of time spent lying in contact with each other as the calves got older (*p* < 0.001).

## 4. Discussion

This study compared the effects of individual and pair housing of pre-weaning calves on their health and behaviour when managed on a single commercial dairy farm to establish if existing research findings were applicable under UK management and environmental conditions. Although this study was performed on a single farm, the management system used adhered to guidelines stipulated by Red Tractor “https://redtractor.org.uk/ (accessed on 30 April 2023)”, which covers around 95% of UK dairy farms, and Arla “https://news.arlafoods.co.uk/sustainable (accessed on 30 April 2023)”, which covers around 27% of UK dairy farms. Therefore, we feel that the findings from this study will be representative of UK commercial enterprises. The single farm set-up also ensured all management and environmental conditions remained the same for both housing groups, along with the large sample size allowing for the detection of significant differences within the study.

### 4.1. Mortality

The overall pre-weaning calf mortality in this herd was 2.8% (13/457), which is in agreement with that found by [46] in the UK and by [47] in Ireland. However, it is lower than that reported in UK national records, 3.9%, although this analysis included both male and beef calves, which are known to have higher mortality levels than replacement dairy heifers [48]. 

### 4.2. Birthweight and Weight Gain

Calf weights were estimated by a single operator using a weigh tape. Such readings are highly correlated with actual weight measurements (r^2^ = 0.89), [49] although others have reported that tapes are less reliable for measurements of ADLG made over short (<3 week) time intervals [50]. The use of a calibrated weighing scale would have offered a gold standard method for weight measurements, but is a more stressful procedure for the calves as it requires moving them out of their pens. Despite this challenge, we found that heavier-birthweight calves demonstrated significantly increased ADLG, with a 1 kg heavier birthweight resulting in an estimated 9.0 g/day increase in ADLG, similar to the findings of [20]. This supports the conclusions reached by [51] that higher-birthweight calves have a greater capacity for pre-pubertal growth, independent of breed. 

The overall ADLG was 0.72 kg/day, with no difference between housing groups (*p* = 0.76), which is in agreement with multiple other studies [19,21,22,52,53]. Although some studies have reported improved growth rates in pair-housed calves [12,13,14], the likely reason for not finding this here was the restricted milk feeding diet that these calves received (6 L of milk per day). Even on low-milk diets, young calves do not consume large quantities of concentrate feed, with a rumen that is not yet developed enough to allow digestion of what is ingested. This means calves are not able to make up energy deficits from low milk intakes via concentrate intakes in the early pre-weaning period, resulting in very low ADLG early in life (Figure 1). However, we did find a trend of pair-housed calves having higher ADLG from 7–8 weeks of age, suggesting that social facilitation increased solid feed intake in pair-housed calves. Continuing to monitor growth after the weaning period would have allowed further investigation into the longer-term impact of this trend on ADLG difference.

### 4.3. Passive Transfer and Disease Occurrence

Calves were given two 3 L feeds of colostrum, which was harvested within 1 h of parturition from the dam, and tested with a brix refractometer prior to feeding. This ensured excellent passive transfer, with 95% of calves demonstrating TP levels ≥ 5.2 g/dL. Despite this, 34.7% of calves experienced some form of disease, with a higher incidence in the individually housed than pair-housed calves (41.5% vs. 30.7%). A similar conclusion was reached by [54], even though individual and pair-housed calves had similar levels of antibody expression when exposed to a novel protein. This contrasts, however, with other earlier studies, which demonstrated no difference in disease occurrence between the housing groups [18,21,22,55]. This may be due to smaller samples sizes than the present study, along with four of these published papers being conducted in research institutions, which may present lower disease risks than commercial enterprises. There is evidence for higher stress levels in individually housed calves, as shown by behaviours like increased vocalisation [13,19], with a lack of social companions shown to increase circulating cortisol levels. Stress negatively impacts the immune response [56] by stimulating the hypothalamic–pituitary–adrenocortical (HPA) axis [57]. This can cause long-term downregulation of immune functions [58], lowering an individually housed calf’s ability to respond to disease pressures effectively [59]. It should be noted that the sampling frequency for disease detection (weekly) may have resulted in some occurrences being missed. However, the researchers feel that this would have impacted all calves equally; therefore, any under-detection of disease should have had a similar impact between housing groups.

### 4.4. Activity

The mean lying time of calves was 18 h 11 min/d, which is in agreement with [17], but slightly longer than that reported by [22] of just over 17 h. Informal observation noted that the predominant periods of activity were around feeding times in the morning and afternoon, which has been reported by others [23]. Summer-born calves (July and August) had approximately 30 min longer lying times than autumn-born calves (September and October). Heat stress generally leads to decreased calf lying times [60,61,62,63], but this is inverse to the pattern seen here, so the reason for this difference is unclear. Lying time was also impacted by birthweight, with heavier newborn calves found to have reduced lying times over the whole pre-weaning period, although there was only a 2 min difference, which may not be biologically significant.

Although there was no significant effect of housing group on lying times, the pair-housed calves were more active than the individually housed calves, as demonstrated by the motion index (MI). Given that the MI is a measure of the intensity of movement and can be used to identify play behaviours in calves [64], these findings suggest that pair-housed calves experienced more positive emotions, with play behaviour indicating good welfare [65]. Space availability will have impacted this, as although the space per calf was the same within both housing groups, pair-housed calves had a higher total housing area. Play behaviour requires sufficient space to occur [65], so this may have been why pair-housed calves were more active [22].

Calves experiencing disease had significantly increased lying times compared to their healthy counterparts (18 h 19 min vs. 18 h 6 min). This was mirrored by a trend of reduction in activity in diseased compared to healthy calves. This may have been due to energy conservation, or potential disease-induced depression leading to reduced activity [66]. Other research has demonstrated no difference in lying times, but a reduction in lying bouts due to disease [32,67,68] or even decreased lying times due to abdominal discomfort from neonatal diarrhoea [69]. The predominant disease occurring in this study was BRD; therefore, it is possible that respiratory as opposed to enteric disease could have led to these increased lying times.

### 4.5. Behaviour

Previous research has suggested that individually housed calves show more fear and reluctance to approach objects when the calves are placed into a novel environment [17,70]. In this study, however, the novel object was placed within the calf’s own environment, and individually housed calves approached more rapidly than the pair-housed calves [71]. This agrees with [72,73], who found that individually housed calves had shorter latencies to contact a human compared to pair-housed calves when they entered the calf’s own pen. This might suggest that individually housed calves are keen to investigate novelty within their own familiar environment, possibly seeking stimuli to replace the lack of social contact [70]. This is supported by individually housed calves spending significantly more time with their head out of the front of the pen, which agrees with [22], and suggests that individuals were attempting to interact with either other calves or stock people. This behaviour increased as the calves got older. It is also worth noting the large difference in novel object approach times between Jersey and Holstein breed calves, with Jerseys having a very short latency to approach (mean 11.8 ± 2.4 s), possibly due to the inquisitive nature of this breed.

Non-nutritive oral behaviours (NNOBs) were entirely focused towards inanimate objects in individually housed calves, whereas in pair-housed calves they also included sucking, licking or chewing behaviours on the body of their pen companion (Table 1). Interestingly, there was no significant difference in the total time engaged in NNOBs between housing groups, with the pair-housed calves spending over half of their NNOB time directed towards inanimate objects, and the remaining time directing NNOBs towards their pen mate. Many descriptions of NNOBs have been focused around the ventrum (including the umbilical area, prepuce and scrotum), as this is where the udder of the dam would be, but others have reported NNOBs directed to differing body parts such as the nose and ears, possibly due to them already having milk on them from the feeding process [74,75]. This was also found in this study, with a mean of 8.5 min of NNOB directed at the ventrum and 12 min directed towards other areas of the body (Table 3). This distribution across the body should help reduce any risks associated with udder health, namely blind quarters or intersucking (sucking by a heifer on her pen mate’s udder), which is sometimes seen in lactating heifers [76], although there is little evidence to suggest that pre-weaning NNOBs are a significant risk for these occurring [77].

NNOBs are mainly driven by a calf’s motivation to suckle, which is stimulated by the ingestion of milk [74,78]. Calves receiving restricted milk feeding diets, like in this study, are therefore at an increased risk of developing NNOBs, especially when the teat bucket used for feeding is removed after milk consumption is complete. Leaving a teat in place for at least 15 min following the end of milk consumption does reduce NNOBs [75,79], and could be used as a mitigation technique if farmers are concerned about the level of NNOBs in pair pens. It was also noted that the total time engaged in NNOBs increased by 17 min per day between one and five weeks of age, regardless of housing type (Table 3). This may have been linked to the restricted milk feeding, meaning as calves got older, the proportion of their diet satisfied by the milk meal decreased, leaving calves unsatiated. The calves also had the same teat throughout the pre-weaning period, so there was the potential for the size of the hole in the teat to enlarge over time, allowing for more rapid ingestion of the milk feed, which is a risk factor for calves engaging in NNOBs [80].

Within our data, it was not possible to distinguish NNOBs directed at the body of a calf from allogrooming, which is when calves groom each other. Pair-housed calves do engage in social activities like allogrooming, with solicited licking often directed towards the head and neck regions, and unsolicited licking occurring around the back and rump regions [81]. Within our data, this may mean that some of the NNOBs, especially those directed to parts of the body other than the ventrum, were actually social grooming, which can be seen as a positive interaction due to its reported cleaning, tension-reducing and social bonding effects [81,82]. These positive grooming behaviours could carry forward as the calves become adults, with allogrooming within the dairy herd occurring more between individuals that are close in birth date that exhibit kinship [83,84].

Time spent self-grooming was significantly associated with the housing group, with individually housed calves spending a mean of 14 min more per day engaged in this activity, which is in agreement with findings from [14,53]. Self-grooming forms part of the normal care of an animals’ own body, but increased levels in individually housed calves can be interpreted as a way for calves to satisfy their need for socialisation. Such displacement behaviour can be elicited by placing cattle in stressful situations, and has been used as a measure of behavioural responsiveness [85]. This suggests that more self-grooming in individually housed calves could be due to increased stress. One role of self-grooming is to clean the hair coat, leading to potential ingestion of faecal material, with both Salmonella type colonies and Coliforms being isolated from the coats of calves [30]. This could be linked to the increased disease prevalence seen in the individually housed calves, as well as increasing the numbers of hair balls reportedly found in individually housed calves [23].

Finally, pair-housed calves were found to spend most of their joint lying time located within one calf’s length of each other, along with a significant increase in the number of lying bouts touching each other as they got older. This is in contrast to the findings of [86] that younger calves may seek security and thermal comfort from each other more than older calves. It is known that calves with stronger social bonds tend to lie closer together [84], so it may be that by five weeks of age, the social bond between the calves was stronger. There may also be an impact of the size of the calf relative to the pen area, with older, larger calves having relatively less room, resulting in increased proximities to each other [87].

## 5. Conclusions

This study aimed to assess the impact of individual and pair housing of pre-weaning calves in a commercial UK farming system. Overall, there were many similarities with the previously published literature from other countries and research centres, with no impact of housing type on ADLG, likely due to a restricted milk feeding diet. We found an increase in disease prevalence in individually housed calves, which may have been due to the negative impacts that stress from social isolation can have on the immune system. There was an increase in the activity of pair-housed calves, which may indicate more play behaviours in social groups, and is an indicator of positive welfare. We also found that both housing groups spent similar total times exhibiting non-nutritive oral behaviours, but individually housed calves spent more time with their head out of the front of the pen, and also spent longer engaged in self-grooming, which agrees with other work. Overall, pair housing had a positive impact on the health and behaviour of calves on this UK commercial dairy farm.

## Figures and Tables

**Table 1:** Description of social interaction and point-based recorded behaviours for calves from video footage. NNOB is a non-nutritive oral behaviour.

Behaviour	Description
NNOB in paired calf on ventral region	The focal calf’s head is under the belly/ventral aspect of another calf and it is sucking, licking or chewing on the mammary or umbilical area of the receiving calf [42].
NNOB in paired calf on body part other than in the ventral region	The focal calf is sucking, licking or chewing on the skin of body parts other than the mammary or umbilical area of the receiving calf [42,43].
NNOB on inanimate object	Focal calf is sucking, licking or chewing on the walls of the pen [43,44].
Calf with head out of the front of the pen	Focal calf has its head (defined as the level of both eyes) through a gap in the front of the pen. The calf could be drinking, eating concentrates or observing outside of the pen.
Self-grooming	Focal calf is grooming, licking or scratching its own body whilst in the standing position [21,44].
Lying proximity of pair calves	Both calves in a pair are lying (body contact with the ground [45]). Proximity defined as touching, <1 calf length apart, 1–2 calf lengths apart.

**Table 2:** Causes of mortality of the individually and pair-housed calves.

Causes of Mortality	Individual (%)	Pair (%)
Joint ill	1/171 (0.6)	1/286 (0.3)
Broken leg	1/171 (0.6)	0/286 (0.0)
Dislocated hip	1/171 (0.6)	2/286 (0.7)
Unknown cause	4/171 (2.3)	3/286 (1.0)
Overall	7/171 (4.1)	6/286 (2.1)

**Table 3:** Disease prevalence between the individually and pair-housed calves.

Disease Prevalence	Individual (%)	Pair (%)
BRD ^1^	44/164 (26.8)	57/280 (20.4)
Diarrhoea	22/164 (13.4)	26/280 (9.3)
Navel ill	2/164 (1.2)	2/280 (0.7)
Joint ill	0/164 (0)	1/280 (0.4)
Overall	68/164 (41.5)	86/280 (30.7)

^1^ BRD: bovine respiratory disease.

**Table 4:** Summary of the minutes per day spent (±standard deviation) on the recorded behaviours of individually and pair-housed calves.

Behaviour	Individually Housed Calf (min)		Pair-Housed Calf (min)	
	1 Week Old	5 Weeks Old	1 Week Old	5 Weeks Old
Calf with head out of the front of the pen	44 ± 26	63 ± 15	24 ± 14	41 ± 13
Self-grooming	28 ± 17	38 ± 15	11 ± 8	26 ± 11
Total NNOB	31 ± 13	48 ± 12	34 ± 23	51 ± 17
NNOB on inanimate object	31 ± 13	48 ± 12	14 ± 11	29 ± 14
NNOB on paired calf on ventral region	NA	NA	9 ± 9	8 ± 5
NNOB on paired calf on body part other than in the ventral region	NA	NA	11 ± 15	13 ± 8

NNOB, non-nutritive oral behaviour; NA, not applicable.

**Table 5:** Summary of the lying positions of the pair-housed calves in relation to each other (±standard deviation), with the *t*-test significance between the two time points shown. Distances between calves were only measured when both calves were lying down.

Lying Position	Pair-Housed Calf (min)		*p*-Value
	1 Week Old	5 Weeks Old	
Touching	3 ± 1	8 ± 3	0.001
<1 calf length apart	17 ± 3	17 ± 4	0.92
1–2 calf lengths apart	11 ± 4	9 ± 5	0.32

## Data Availability

Data is available on request to the corresponding author.
